# Antioxidant and Hepatoprotective Effects of Silibinin in a Rat Model of Nonalcoholic Steatohepatitis

**DOI:** 10.1093/ecam/nep164

**Published:** 2011-01-12

**Authors:** Yara Haddad, Diane Vallerand, Antoine Brault, Pierre S. Haddad

**Affiliations:** ^1^Natural Health Products and Metabolic Diseases Laboratory, Department of Pharmacology and Montreal Diabetes Research Center, Université de Montréal, Montréal, QC, Canada H3C 3J7; ^2^Institute of Nutraceutical and Functional Foods, Laval University, 2900 Boulevard Edouard Montpetit, Québec City, QC, Canada H3T 1J4

## Abstract

Nonalcoholic steatohepatitis (NASH) is a progressive liver disease related to the metabolic syndrome, obesity and diabetes. The rising prevalence of NASH and the lack of efficient treatments have led to the exploration of different therapeutic approaches. Milk thistle (*Silibum marianum*) is a medicinal plant used for its hepatoprotective properties in chronic liver disease since the 4th century BC. We explored the therapeutic effect of silibinin, the plant's most biologically active extract, in an experimental rat NASH model. A control group was fed a standard liquid diet for 12 weeks. The other groups were fed a high-fat liquid diet for 12 weeks without (NASH) or with simultaneous daily supplement with silibinin–phosphatidylcholine complex (Silibinin 200 mg kg^−1^) for the last 5 weeks. NASH rats developed all key hallmarks of the pathology. Treatment with silibinin improved liver steatosis and inflammation and decreased NASH-induced lipid peroxidation, plasma insulin and TNF-**α**. Silibinin also decreased O_2_^∙−^ release and returned the relative liver weight as well as GSH back to normal. Our results suggest that milk thistle's extract, silibinin, possesses antioxidant, hypoinsulinemic and hepatoprotective properties that act against NASH-induced liver damage. This medicinal herb thus shows promising therapeutic potential for the treatment of NASH.

## 1. Introduction

In industrialized countries, a sedentary lifestyle and visceral adiposity are the main factors underlying the development of steatosis, a benign accumulation of hepatic lipids and part of the nonalcoholic fatty liver disease (NAFLD) spectrum. However, nonalcoholic steatohepatitis (NASH), which is the most serious form of NAFLD, is more likely to evolve toward fibrosis and cirrhosis. Its prevalence has exhibited a sharp rise reaching approximately 3% of the general population [1]. The progression from steatosis to NASH is a numerous-hit process that is usually achieved with the participation of dominant phenomena such as insulin resistance, oxidative stress and inflammation [2, 3]. Although the exact pathogenesis of NASH is yet to be understood, several studies have focused on strongly related markers such as lipid peroxidation, reactive oxygen species (ROS) production, mitochondrial dysfunction, secretion of inflammatory cytokines and adipokines, infiltration of polymorphonuclear leukocytes, hepatocyte ballooning, formation of Mallory bodies and collagen deposition to confirm the diagnosis of NASH or to develop new treatment strategies [2–5]. Among these strategies, the use of complementary and alternative medicine approaches, such as natural antioxidants and hepatoprotective plant products, has been gaining popularity in the last decade.

Milk thistle (*Silibum marianum*) is the most ancient and extensively used medicinal plant for its beneficial effects on liver and other organs [6]. It was primarily recognized for its capacity to enhance bile flow and to remove obstructions from liver and spleen [6, 7]. Silymarin, a flavolignan extracted from the fruits and seeds of the plant, was found effective for protecting against cirrhosis, jaundice and hepatitis [7]. Furthermore, *in vitro*, *in vivo* and clinical studies have demonstrated the antioxidant and hepatoprotective effects of silymarin and its major active constituent silibinin (silybin), a polyphenolic molecule, in animal and human models of alcoholic or nonalcoholic chronic liver diseases [8–14]. *Silibum marianum* is typically administered as an encapsulated standardized extract (70–80% silymarin) that is poorly absorbed. However, studies carried out in rats and humans have proven the superior bioavailability of silibinin when complexed with phosphatidylcholine (Siliphos, Silipide) [15–18]. Indeed, the hepatoprotective and antifibrotic effects of a silibinin–vitamin E–phospholipids complex in a rat model of chronic liver disease were demonstrated by Di Sario and colleagues [19]. Finally, a recent pilot study has also illustrated the effectiveness of the complex in reducing insulin resistance and plasma markers of liver fibrosis in patients with NAFLD [20]. However, no detailed analysis of the beneficial effect and mode of action of silibinin, whether alone or as a complex, has ever been reported in the context of NASH. Thus, the present study was conducted with the objective of evaluating the efficacy of the silibinin–phosphatidylcholine complex in protecting the liver against diet-induced NASH in rats.

## 2. Methods

### 2.1. Chemicals

All chemicals were purchased from Sigma Aldrich (St-Louis, USA). Silibinin was obtained from the commercially available silibinin–phosphatidylcholine complex (Siliphos, Silipide).

### 2.2. Animals and Experimental Procedure

A total of 20 Sprague-Dawley male rats, each weighing about 75–100 g, were purchased from Charles River, Canada (St-Constant, Quebec). They were acclimatized to our animal care facilities for 5 days, during which they had free access to normal rat chow diet and water before the beginning of experimental protocols. Rats were then randomly allocated to three groups. The Control group comprised of six rats that were fed with the Lieber–DeCarli standard liquid diet for 12 weeks. The NASH group, also comprised of six rats, received the Lieber–DeCarli high-fat liquid diet for 12 weeks. The treatment group (Silibinin) comprised of eight rats that were fed with the same high-fat diet for 12 weeks to which was added the silibinin–phosphatidylcholine complex, at a dose of 200 mg kg^−1^ (as silibinin) daily, during the last 5 weeks. Preliminary experiments confirmed that cardinal parameters of NASH were present after 7 weeks of high-fat diet as compared with the Control group—(histology 75% of hepatocytes at stage 2 of steatosis and level 2 of inflammation versus 100% of hepatocytes at stage and level 0, resp., *n* = 4, chi square *P* < .05); (malondialdehyde, MDA, 2.02 ± 0.25 versus 0.69 ± 0.06 *η*mol mg^−1^ of protein; lactate dehydrogenase, LDH, 95.0 ± 11.1 versus 68.3 ± 12.1 U L^−1^; ATP 3.43 ± 1.22 versus 6.37 ± 0.60 *μ*mol g^−1^ of protein; liver weight, LW, 15.8 ± 0.7 versus 12.8 ± 0.6 g, resp.; *n* = 4, *P* < .05)—at the time when silibinin treatment was initiated. Diets were acquired from Dyets Inc. (Bethlehem, PA) and mixtures were freshly prepared daily. The respective energetic balances of each diet are listed in Lieber et al. [21]. Animals were fed *ad libitum* and kept in a room with controlled temperature and humidity, under a 12:12 h light–dark cycle. They were monitored weekly for weight and food consumption. All experimental protocols were approved by the University's Ethics Committee and accomplished in accordance to the guidelines of the Canadian Council on the Care of Animals.

### 2.3. Liver Isolation

Surgical procedure was performed after overnight fasting, as described by Huet et al. [22]. Rats were anesthetized with an intraperitoneal injection of sodium pentobarbital (50 mg kg^−1^ body weight, BW). Rats underwent laparotomy and the portal vein was cannulated. Blood was collected via the inferior vena cava and stored as plasma and serum. Glycemia was directly evaluated using a commercial glucometer (One Touch Ultra, LifeScan, Johnson & Johnson, Milpitas, CA). Livers were flushed for 3 min with Krebs–Henseleit buffer (NaCl 145 mM, KCl 4.8 mM, MgSO_4_ 1.2 mM, NaHCO_3_ 25 mM, CaCl_2_ 2.1 mM, pH 7.4, 22°C) then removed and weighed. Liver dissection was subsequently performed. Sections from the median lobe were placed in a 10% formalin solution for histopathological analysis and other samples were immediately frozen in liquid nitrogen and stored at –80°C until use.

### 2.4. Histology

Hematoxylin phloxine saffron (HPS) staining was performed on histological sections by the Department of Histology, St-Justine's Children Hospital (Montreal, Qc, Canada). Scoring for steatosis was based on the percentage of hepatocytes containing macrovesicular fat in each section (grade 0 absent; grade 1 < 33%; grade 2 33–66%; grade 3 >66%), according to published criteria [23]. The degree of inflammation was based on the number of immune cells encountered in 10 randomly selected areas (at ×400) within each section using the following scale (0 normal; 1 mild; 2 moderate; 3 severe) [23]. Mallory bodies and collagen deposition were evaluated as present or absent. Photos were taken using Axio-imager electronic microscope and Axio-vision 4.2 software (z1 Zeiss, Jena, Germany).

### 2.5. Blood Parameters

Plasma lipids, namely triglycerides (TG), total cholesterol (TC), low-density lipoprotein (LDL), high-density lipoprotein (HDL) as well as transaminases (alanine aminotransferase, ALT, and aspartate aminotransferase, AST) and LDH were measured by the Department of Biochemistry, St-Justine's Children Hospital (Montreal, Qc, Canada). Plasma tumor necrosis factor-*α* (TNF-*α*) was assessed using Quantikine Rat TNF-*α* Immunoassay Kit (R&D Systems, MN, USA). Serum adiponectin was measured with a Rat Adiponectin ELISA Kit (AdipoGen, Seoul, Korea). Plasma insulin was evaluated using a rat insulin radioimmunoassay (RIA) kit (Linco Research, St. Charles, MO). The Homeostasis Model Assessment of Insulin Resistance (HOMA-IR) index was calculated using circulating insulin and glucose values with the following formula: (Fasting insulin (mU L^−1^) × fasting blood glucose (mmol L^−1^])/22.5 [24].

### 2.6. Isolation of Mitochondria

Fresh liver (1 g) was used for the isolation of mitochondria, as described by Johnson and Lardy [25]. Briefly, an ice-cold Teflon potter was used to homogenize tissue in the isolation medium containing sucrose (250 mM), Tris–base (10 mM), Ethylene glycol tetraacetic acid (EGTA) (1 mM), pH 7.2 at 4°C. The homogenate was then centrifuged at 600 g for 10 min at 4°C, in order to remove cellular fragments. The supernatant was collected and centrifuged at 15 000 g for 5 min at 4°C. The resulting pellet was subsequently washed gently with the same medium, centrifuged and washed another time with the isolation medium without EGTA and centrifuged one last time. The final pellet, which contained the viable mitochondria, was suspended in this last medium and stored on ice. Protein content was determined following the Lowry method [26].

### 2.7. Assessment of Oxidative Stress Parameters

The thiobarbituric acid (TBA) method was used to evaluate liver lipid peroxidation by measuring MDA levels, based on Ligeret et al. [27]. Briefly, fresh liver (0.5 g) was homogenized on ice in a sucrose buffer (4.5 mL, 250 mM) using a Teflon potter homogenizer and a polytron. The homogenate was centrifuged at 2000 g for 30 min at 4°C. The protein content of the supernatant was determined using the Bradford method. The supernatant (200 *μ*L) was added to a vial containing an 8.1% (w/v) sodium dodecyl sulfate solution (200 *μ*L), a 20% (v/v) acetic acid solution (pH 3.5) (1.5 mL), a 0.8% (w/v) TBA solution (1.5 mL) and distilled water (600 *μ*L). Vials were heated at 95°C for 45 min and cooled on ice for 2 min. Butanol (4 mL) was added and all vials were mixed for 15 min and then centrifuged at 1000 g for 10 min. Finally, 200 *μ*L of supernatants were transferred into a 96-well plate and Thiobarbituric Acid Reactive Substances (TBARS) were estimated by measuring fluorescence (*λ*_ex_ = 530 nm; *λ*_em_ = 590 nm) using a multilabel counter model Wallac Victor^2^ (Perkin Elmer, Woodbridge, ON). The data were compared with a standard curve of MDA (0–84 *μ*M) and results were expressed as nmol MDA mg^−1^ of protein.

The superoxide anion (O_2_^∙−^) in liver tissue was determined using the Lucigenin-amplified chemiluminescence procedure, as described by Oliveira and colleagues [28]. In brief, a frozen liver fragment was incubated for 15 min in an oxygenated (95% O_2_ and 5% CO_2_) Krebs-HEPES buffer containing NaCl (118.3 mM), KCl (4.69 mM), CaCl_2_ (1.87 mM), MgSO_4_ (1.2 mM), KH_2_PO_4_ (1.03 mM), NaHCO_3_ (25 mM), glucose (11.1 mM) and Na-HEPES (20 mM), pH 7.4, at 37°C. The fragment was transferred into a scintillation vial containing 2 mL of this buffer, supplemented with lucigenin (250 mM). The scintillation counter (Wallac 1409 Model, Perkin Elmer Life Science, St-Laurent, QC) was adjusted for single-photon emission recording mode. The counts appraisal was performed during a 15-min period. Data were obtained from the area under the counts versus time curve and expressed as a function of the dry tissue's weight (mg).

To assess reduced glutathione (GSH), 0.5 g of fresh liver was minced on ice and then homogenized with an ice-cold polytron, in 1 mL of freshly prepared metaphosphoric acid solution (5%). Homogenate was centrifuged at 3000 g for 10 min at 4°C. The supernatant was used for GSH assessment using the Bioxytech GSH-400 Assay Kit (Oxis International Inc, Portland, USA) according to the manufacturer's instructions. Absorbance at 400 nm was evaluated using an Ultrospec 2100 pro spectrophotometer (Biochrom, Cambridge, England). Data were compared with a standard curve of reduced GSH (0–90 *μ*M).

### 2.8. Mitochondrial ATP

Freshly isolated mitochondria (100 *μ*L) were added to ice-cold HClO_4_ (900 *μ*L, 1 M) and centrifuged at 2000 g for 10 min at 4°C. The supernatant (100 *μ*L) was neutralized with KOH (47 *μ*L, 2 M) and Tris–HCl (853 *μ*L, 100 mM) [27]. Assessment of bioluminescence was carried out using the ATP Bioluminescent Assay Kit (SIGMA, Oakville, ON) according to the manufacturer's instructions with a multilabeled counter model Wallac Victor^2^ (Perkin Elmer, Woodbridge, ON). The data were then compared with a standard curve of ATP (0–80 *μ*M) and results were expressed in *μ*mol ATP g^−1^ of protein.

### 2.9. Western Blotting

Western blot analysis was performed on total liver homogenate, based on Lieber et al. [21], using a rabbit polyclonal anti-PPAR-*α* primary antibody (1 : 400) (Santa Cruz Biotechnology Inc. Santa Cruz, CA) and a goat polyclonal anti-rabbit secondary antibody (1 : 100 000) (Jackson Immunoresearch Laboratories, Baltimore, PA). Liver microsomal fraction was also subjected to western blot analysis using mouse monoclonal anti-rat P450 CYP2E1 primary antibody (1 : 1000) (Oxford Biomedical Research, Oxford, MI) and a goat anti-mouse secondary antibody (1 : 4000) (Cell Signaling Technology, Danvers, MA). Immunoreactive proteins were detected with an enhanced chemiluminescence system according to the manufacturer's instructions (GE Healthcare, ECL Western Blotting Detection Reagents, Buckinghamshire, UK). Band quantification was performed using ImageJ 1.37v (NIH, USA).

### 2.10. Statistical Analysis

All results are expressed as means ± standard error (SE). Group means were compared by one way ANOVA followed by Fisher's PLSD test (and the Chi square test for histological contingency table) using StatView 5.0.1 (SAS Institute Inc. NC). A *P* value <  .05 was considered statistically significant.

## 3. Results

### 3.1. Changes in BW and LW

As shown in Table 1, there were no significant differences in BW or weight gain (WG) between groups. In contrast, the high-fat diet increased the LW in the NASH group as compared with the Control group and this resulted in a significant rise in liver index (LI). Moreover, silibinin treatment during the last 5 weeks of the high-fat diet protocol decreased the LW and the LI significantly when compared with the NASH group (*P* < .05) and brought these parameters back to values very similar to those observed in the Control group.

### 3.2. Glycemic Homeostasis

**Figure 1:**
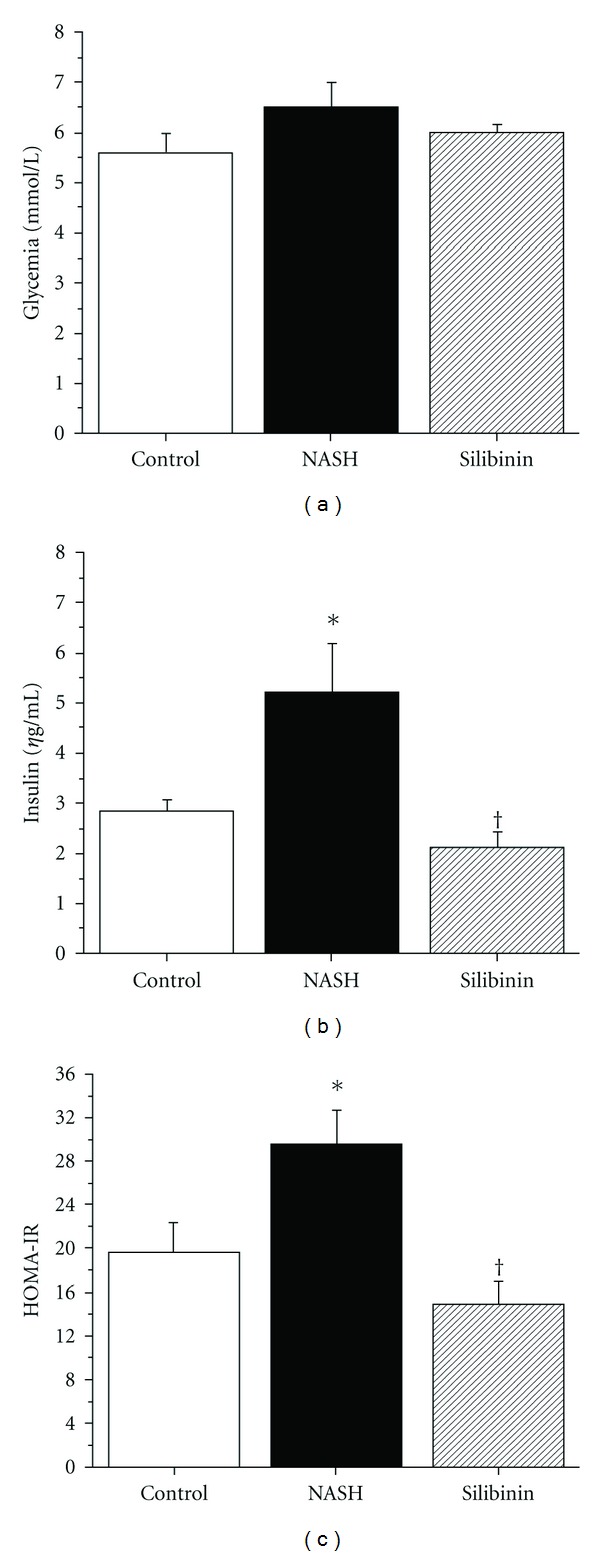
Effect of silibinin on glycemic homeostasis. Glycemia (a) was measured in whole blood and insulin (b) was measured in plasma obtained from rats in the fasting state, after 12 weeks of standard diet (control) or high-fat diet alone (NASH), or supplemented with silibinin–phosphatidylcholine complex (Silibinin), at a dose of 200 mg kg^−1^ as silibinin, during the last 5 weeks. HOMA-IR (c) is indicative of the insulin resistance state for the animals. Values are expressed as means ± SE of six rats for Control and NASH groups and of eight rats for Silibinin group. *P < .05 versus Control group. ^†^ *P* < .05 versus NASH group.

Fasting blood glucose had a tendency to be slightly more elevated in NASH animals than in controls or silibinin-treated congeners, albeit no statistically significant changes were observed (Figure 1(a)). In contrast, 12 weeks of high-fat diet induced statistically significant hyperinsulinemia in NASH rats as compared with control animals (plasma insulin values of 5.22 ± 0.98 versus 2.86 ± 0.21 ng mL^−1^, resp.; Figure 1(b), *P* < .05). This situation was associated with an important increase in insulin resistance, as demonstrated by the significantly higher value of HOMA-IR in NASH animals as compared with control congeners (Figure 1(c); *P* < .05). Both plasma insulin and HOMA-IR values were decreased significantly in Silibinin group as compared with NASH group (Figures 1(b) and 1(c); *P* < .05). Moreover, values in the Silibinin group were even lower than those observed in control animals.

### 3.3. Biochemical Parameters

As presented in Table 2, no significant changes were observed in plasma transaminase (ALT, AST) and TC between experimental groups. The 12-week NASH-inducing high-fat diet was associated with a significant increase in plasma LDH and LDL and a significant decrease in TG as compared with controls. HDL also tended to be higher in NASH than in control animals. Silibinin treatment significantly decreased HDL and LDH levels as compared with the NASH group. It also improved LDL, albeit ANOVA analysis failed to reach statistical significance.

### 3.4. Histological Evaluation

**Figure 2:**
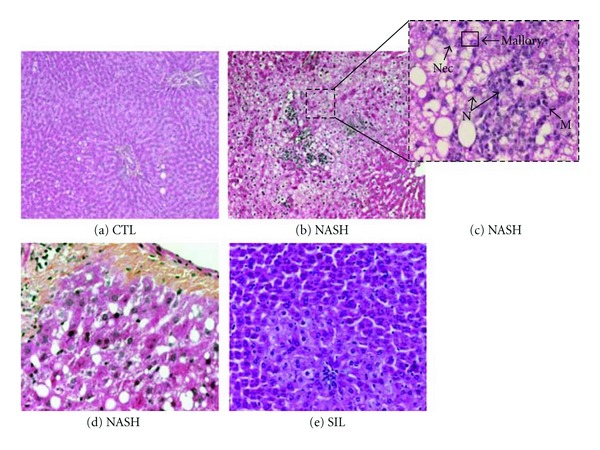
Effect of silibinin on liver histology. Representative HPS staining of rat liver sections. The Control (a-CTL; ×20) group received 12 weeks of standard liquid diet and showed normal histology. The NASH (b–d NASH; ×10, ×40 and ×20, resp.) group received a high-fat liquid diet during 12 weeks and revealed severe steatosis, hepatocyte ballooning, important inflammatory cell infiltration such as monocytes (M) and neutrophils (N), necrosis (Nec), Mallory bodies (Mallory) and fibrosis. A 12-week high-fat liquid diet supplemented with silibinin–phosphatidylcholine complex (e-SIL; ×20), at a dose of 200 mg kg^−1^ as silibinin during the last 5 weeks, displayed mild steatosis and moderate inflammation.

Liver biopsy evaluation is presently the gold standard test for the diagnosis of NASH. In the Control group, liver sections exhibited normal global histological features (CTL in Figure 2(a) and Table 3). Liver sections from rats in the NASH group revealed that more than one-third of hepatocytes contained macrovesicles of fat (NASH in Figure 2(b)), which corresponded to grade 2 or 3 of steatosis (Table 3). In addition, necrotic hepatocytes, Mallory bodies (NASH in Figure 2(c)) and collagen deposition (NASH in Figure 2(d)) were exclusively found in samples from the NASH group. A 5-week treatment with 200 mg kg^−1^ of silibinin promoted significant improvement in histology (SIL in Figure 2(e)). Indeed, >60% of rats in the Silibinin group had less than one-third of hepatocytes containing macrovesicular fat (grade 0 or 1 steatosis), as compared with rats in the NASH group (100% grade 2 or 3 steatosis). In parallel, the liver infiltration of immune inflammatory cells was very rarely encountered in control animals. In contrast, 80% of NASH rats revealed inflammatory cell infiltration ranging from moderate to severe (NASH in Figure 2(c)). Silibinin significantly improved this parameter (Table 3), albeit not back to levels observed in control animals.

### 3.5. Inflammatory Cytokines and Adipokines

**Figure 3:**
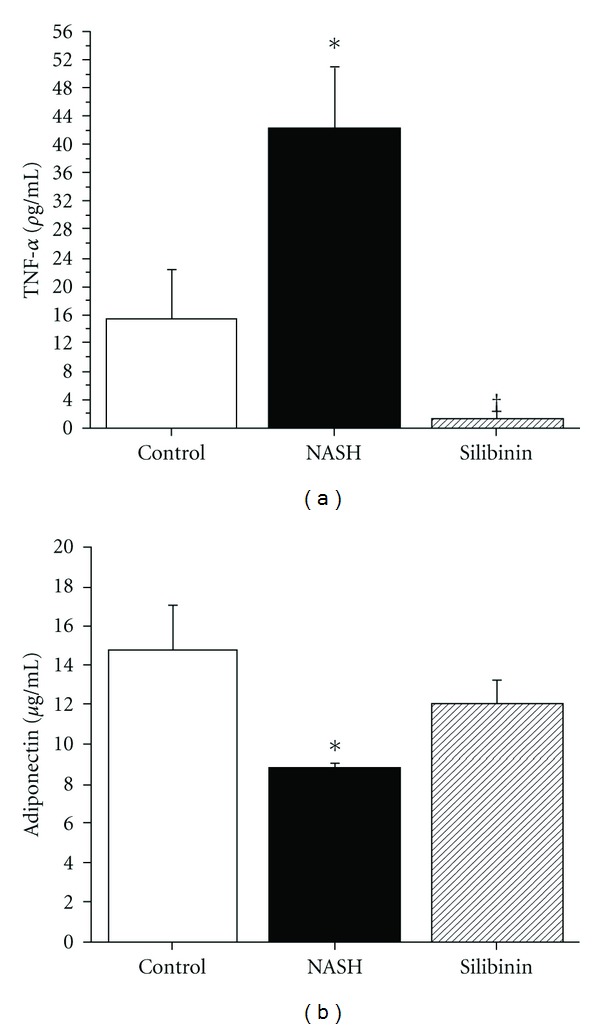
Effect of silibinin on inflammatory cytokines and adipokines. TNF-*α* (a) and adiponectin (b) were measured using ELISA kits in plasma and serum, respectively, and were obtained from rats in the fasting state, after 12 weeks of standard diet (control), high-fat diet alone (NASH) or supplemented with silibinin–phosphatidylcholine complex (Silibinin), at a dose of 200 mg kg^−1^ as silibinin, during the last 5 weeks. Values are expressed as means ± SE of six rats for control and NASH groups and of eight rats for Silibinin group. *P < .05 versus control group. ^†^ *P* < .05 versus NASH group.

Adipose tissue and macrophages (including liver Kupffer cells), produce pro-inflammatory cytokines such as TNF-*α*. During stress, the generation of elevated amounts of TNF-*α* impairs insulin signaling, leading ultimately to insulin resistance. TNF-*α* was higher in the plasma of NASH rats than in that of control rats (42.3 ± 8.8 and 15.4 ± 7.1 *ρ*g mL^−1^, resp.; Figure 3(a), *P* < .05). Conversely, silibinin remarkably and significantly diminished TNF-*α* level (*P* < .05). Another adipokine secreted by the adipose tissue is adiponectin. This hormone is known to decrease during fatty liver disease and the metabolic syndrome. The high-fat diet significantly reduced adiponectin levels in NASH rats as compared with control animals (*P* < .05). The silibinin treatment caused a considerable rise in circulating adiponectin but statistical significance was not achieved (Figure 3(b)).

### 3.6. Oxidative Stress

**Figure 4:**
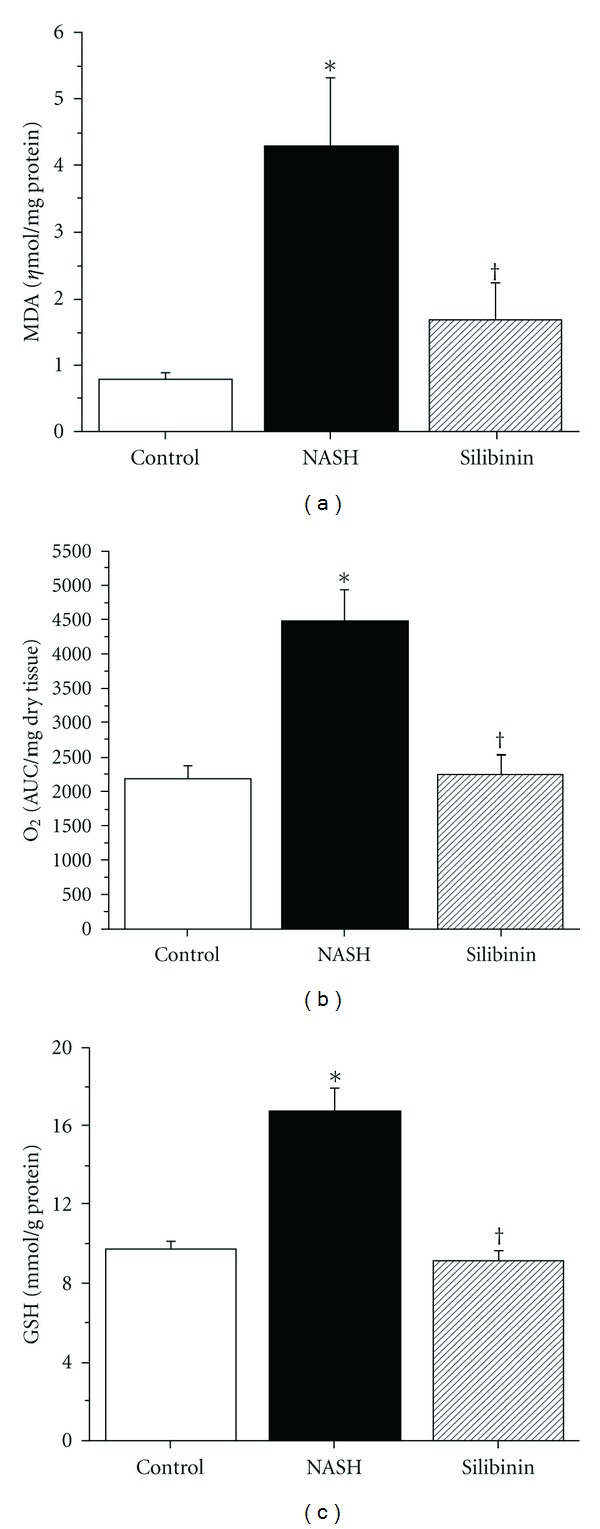
Effect of silibinin on oxidative stress parameters. Hepatic MDA (a), O_2_^∙−^ production (b) and GSH concentration (c) were measured in fresh or frozen liver tissue obtained from rats in the fasting state, after 12 weeks of standard diet (control), high-fat diet alone (NASH) or supplemented with silibinin–phosphatidylcholine complex (Silibinin), at a dose of 200 mg kg^−1^ as silibinin, during the last 5 weeks. Values are expressed as means ± SE of six rats for control and NASH groups and of eight rats for Silibinin group. *P < .05 versus Control group. ^†^ *P* < .05 versus NASH group.

MDA is a lipid peroxidation product generated during oxidative stress. As Figure 4(a) illustrates, the NASH group exhibited a >4-fold increase in hepatic MDA level as compared with the Control group (4.29 ± 1.05 versus 0.80 ± 0.09 *η*mol mg^−1^ of protein, resp.; *P* < .05). Lipid peroxidation was efficiently counteracted by the treatment with 200 mg kg^−1^ of silibinin as compared with the NASH group (*P* < .05).

Also during oxidative stress, the liver produces the superoxide anion (O_2_^∙−^), an important ROS. As shown in Figure 4(b), hepatic O_2_^∙−^ level was nearly twice as high in the NASH group as compared with the Control group (4473 ± 466 and 2197 ± 190 AUC mg^−1^ dry tissue, resp.; *P* < .05). Silibinin was able to significantly reduce O_2_^∙−^ to 2267 ± 267 AUC mg^−1^ dry tissue (*P* < .05 versus NASH animals), a value very similar to that of the Control group.

Hepatic reduced GSH is a potent antioxidant, also produced by the liver as a mechanism of intracellular defense. As illustrated by Figure 4(c), GSH level was significantly higher in the NASH group than in the Control group (16.8 ± 1.2 versus 9.8 ± 0.4 mmol g^−1^ of protein, resp.; *P* < .05). The silibinin treatment significantly diminished hepatic glutathione to a value of 9.3 ± 0.4 mmol g^−1^ of protein, which is equivalent to that of the Control group.

### 3.7. Mitochondrial Dysfunction

**Figure 5:**
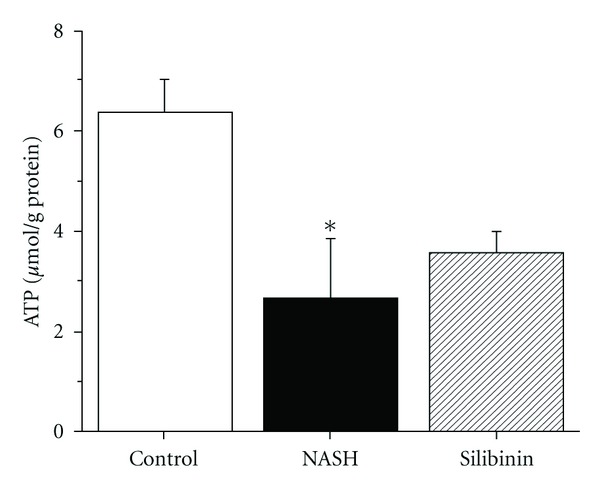
Effect of silibinin on mitochondrial ATP. ATP was measured in freshly isolated mitochondria obtained from rats in the fasting state, after 12 weeks of standard diet (control), high-fat diet alone (NASH) or supplemented with silibinin–phosphatidylcholine complex (Silibinin), at a dose of 200 mg kg^−1^ as silibinin, during the last 5 weeks. Values are expressed as means ± SE of six rats for control and NASH groups and of eight rats for Silibinin group. *P < .05 versus control group.

The high-fat treatment caused a significant decrease in hepatic mitochondrial ATP production, as observed in the NASH group compared with the Control group (Figure 5; *P* < .05). This cellular energetic imbalance was not corrected by silibinin treatment.

### 3.8. Expression of CYP2E1 and PPAR-*α*

**Figure 6:**
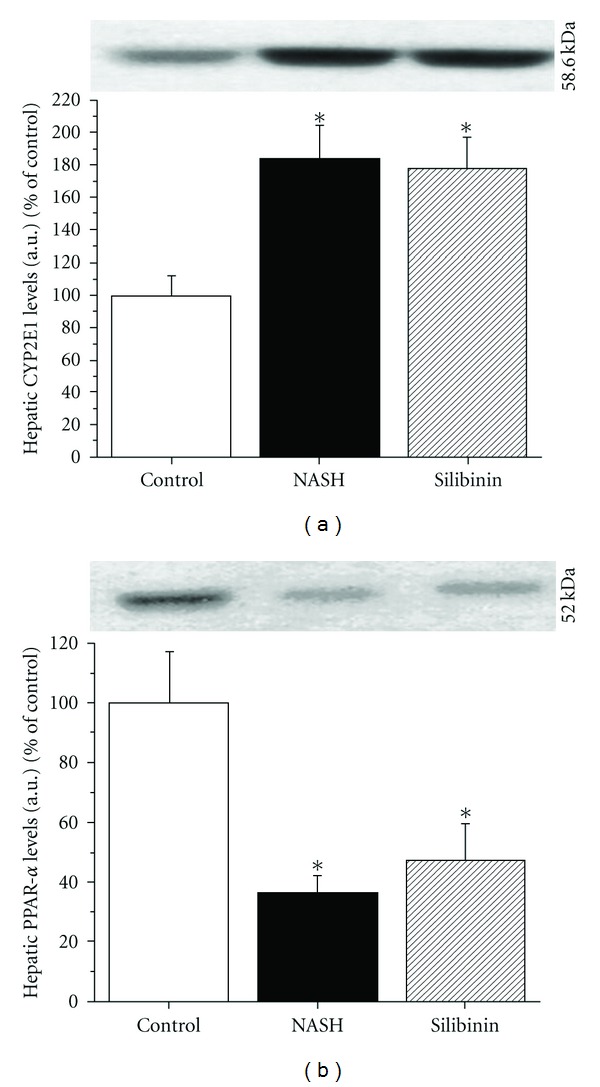
Effect of silibinin on CYP2E1 and PPAR-*α*. Representative western blots presented were performed on liver microsomal fraction for CYP2E1 (a) or whole liver homogenates for PPAR-*α* (b) to reveal their protein expression. Livers were obtained from rats in the fasting state, after 12 weeks of standard diet (CTL), high-fat diet alone (NASH) or supplemented with silibinin–phosphatidylcholine complex (SIL), at a dose of 200 mg kg^−1^ as silibinin, during the last 5 weeks. Values are expressed as means ± SE of 6–8 samples for each group. *P < .05 versus control group.

Impaired mitochondrial *β*-oxidation as well as increased peroxisomal *β*-oxidation and microsomal *ω*-oxidation of free fatty acids (FFAs) in hepatocytes lead to the overproduction of ketones. The cytochrome P450 CYP2E1 is the metabolizing enzyme upregulated by elevated levels of ketone bodies in the liver. As shown in Figure 6(a), the high-fat diet caused an increase in the microsomal expression of cytochrome P450 CYP2E1 in livers of the NASH rats (84% above control, *P* < .05). CYP2E1 expression was unchanged in the Silibinin group as compared with the NASH group.

An important transcription factor responsible for the activation of fat metabolism genes is the peroxisome proliferator-activated receptor-*α* (PPAR-*α*). Its expression was decreased in livers of NASH animals as compared with values observed in the Control group (34% of control, *P* < .05), Silibinin treatment failed to return the expression of PPAR-*α* to control levels (Figure 6(b)).

## 4. Discussion

NASH is a severe subset of NAFLD that may evolve to cirrhosis and liver failure. Its prevalence is increasing in global populations due to the rising incidence of its closely related conditions, namely visceral adiposity, the metabolic syndrome and diabetes mellitus [29]. At present, no pharmacological treatment has been convincingly efficient against NASH. In fact, slight but consistent weight loss, healthy eating regimen and exercise together with a number of therapeutic avenues, remain the center of all strategies to improve or reverse the main NASH-induced injuries [30, 31]. With the rising interest in complementary and alternative medicine, notably in the western hemisphere, several natural products have raised interest for their potential beneficial effects in liver diseases. Among such attractive novel therapeutic possibilities, milk thistle and its major active compounds have caught our interest because of their various properties known, for centuries, to be beneficial in liver disease. These properties, namely antioxidant, hepatoprotective and mitochondrial-protective properties, were also demonstrated in our previous study in which silibinin afforded mitochondrial and antioxidant protection against liver injuries induced by cold preservation/warm reperfusion in rats [27]. Considering that all the aforementioned features contribute to the pathogenesis of NASH, we therefore evaluated the beneficial effects of silibinin in an experimental *in vivo* rat model of NASH.

To induce the pathology, we selected a dietary model developed by Lieber and colleagues [21] that reproduces the dominant clinical features of NASH in rats, following the consumption of a high-fat liquid diet. This model appropriately mimics the human condition given that NASH is intimately associated with a fat-rich sedentary life-style in obese and non-obese patients [32]. Our results clearly demonstrate that the ingestion of the Lieber liquid high-fat diet during 12 weeks produces all the prominent characteristics of NASH. Furthermore, it is important to note that the principal histological features of NASH, including steatosis, inflammation and fibrosis, were found in preliminary experiments to be already present after 7 weeks of the Lieber diet. Therefore, rats received the silibinin therapy during the last 5 weeks of the 12-week experiment with the objective of treating the disease.

Treatment with 200 mg kg^−1^ of silibinin (added to the diet as a silibinin–phosphatidylcholine complex) during these 5 weeks, along with the uninterrupted intake of the high-fat diet, was very efficient at reversing the progression of NASH. Indeed, accumulation of fat in the liver was significantly reduced, as indicated by the improved histological grade of steatosis and by the normalization of liver weight, liver index as well as plasma lipid levels (notably LDL, HDL and TC). Despite this, silibinin treatment failed to restore impaired PPAR-*α* expression or to reduce the elevated expression of CYP 2E1. A reducing effect on CYP 2E1 was previously reported in human hepatocytes but required very high doses of the milk thistle component [33].

On the other hand, insulin sensitivity was restored by silibinin treatment as suggested by normalized insulinemia and HOMA-IR, despite the continued intake of the NASH-inducing high-fat diet. This hypoinsulinemic effect was shown in a previous clinical trial on cirrhotic diabetic patients [12]. There was also a tendency for silibinin to reverse the NASH-induced reduction of circulating adiponectin, consistent with the reversal of insulin resistance. Such an insulin-sensitizing effect was previously observed in patients with chronic liver damage that were treated with a silibinin–vitamin E–phospholipids complex [20]. Part of this effect was attributed to the antioxidant properties of silibinin [12] because of its capacity to decrease lipid peroxidation, to reduce the release of O_2_^∙−^ and to restore hepatic GSH level [34, 35]. Indeed, the latter was evident in the present study from the return to normal of all hepatic oxidative stress parameters (lipid peroxidation, ROS generation, reduced glutathione).

Likewise, the hepatoprotective effect of silibinin was confirmed by the normalized level of circulating LDH and supported by the tendency to restore the NASH-induced loss of mitochondrial ATP. In addition, silibinin treatment significantly improved all indicators of inflammation that were heightened by the induction of NASH. Indeed, the infiltration of immune cells into liver tissue was significantly diminished and circulating TNF-*α* level was remarkably reduced. Moreover, the livers of silibinin-treated rats were devoid of necrosis, Mallory bodies and fibrosis. In fact, silibinin was shown to be antifibrogenic in the liver by reducing activation and proliferation of isolated and cultured stellate cells [36] and by decreasing collagen accumulation in experimental liver fibrosis [37, 38] in rats. The hepatoprotective, antioxidant and antifibrogenic effects of silibinin observed herein are also consistent with previous studies [34, 35, 37–41].

**Figure 7:**
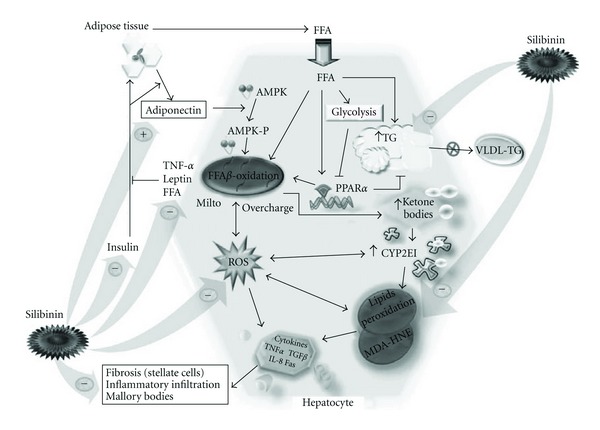
Pathogenesis of NASH and major action fields of the Silibinin treatment. Main results-defined pathological pathway leading to NASH and key action points of Silibinin in the liver to reverse the disease's development. mito, mitochondria; HNE, *trans*-4-hydroxy-2-nonenal; VLDL, very low density lipoproteins; arrow: induces; blunt arrow: inhibits; plus: increases; minus: decreases.

In summary, our understanding of the NASH pathology based on the obtained results leans toward the popular theory of the multiple-hit process leading to cellular and molecular injuries. As mentioned previously, an accumulation of macrovesicular fat accompanied by elevated oxidative stress production within the hepatocytes, along with inflammation and insulin resistance represent the key factors in the development of a steatohepatitis, as illustrated in Figure 7. On the other hand, our results also allow us to speculate on the main points of action of silibinin being steatosis, inflammation, oxidative stress and insulin resistance (Figure 7).

Overall, our study clearly confirms that silibinin, a natural hepatoprotectant, is efficient at improving injuries caused by a chronic liver disease. It also demonstrates for the first time that a therapeutic treatment with silibinin, administrated as a complex with phosphatidylcholine, is effective in reversing steatosis, inflammation, oxidative stress and insulin resistance in an *in vivo* rat model of diet-induced NASH. These results are very promising and, given the fact that silibinin has already been used safely in humans suffering from chronic liver disease, encourage us to propose that this novel therapeutic approach for NASH be tested in future clinical studies.

## Funding

Grant from the Canadian Institutes of Health Research [CTP-79855].

## Figures and Tables

**Table 1:** Effect of silibinin on body and liver weight.

Parameters	BW (g)	WG (g)	LW (g)	LI^a^ (%)
Control	559 ± 18	469 ± 6	14.2 ± 0.5	2.5 ± 0.1
NASH	526 ± 15	433 ± 17	16.7 ± 0.6*	3.2 ± 0.1*
Silibinin	510 ± 12	432 ± 11	13.4 ± 0.8**	2.6 ± 0.1**
ANOVA	0.065	0.085	0.0184	0.0071

Measurements were obtained from rats on the day of sacrifice, after 12 weeks of standard diet (control) or high-fat diet alone (NASH), or supplemented with silibinin–phosphatidylcholine complex (Silibinin), at a dose of 200 mg kg^−1^ as silibinin, during the last 5 weeks. Values are expressed as means ± SE of six rats for control and NASH groups and of eight rats for Silibinin group.

^a^LI, LW/BW × 100.

*P < .05 versus control group. **P < .05 versus NASH group.

**Table 2:** Effect of silibinin on biochemical and lipid parameters.

Parameters	AST (U L^−1^)	ALT (U L^−1^)	TC (mmol L^−1^)	HDL (mmol L^−1^)	LDL (mmol L^−1^)	TG (mmol L^−1^)	LDH (U L^−1^)
Control	57 ± 6	28 ± 3	0.79 ± 0.08	0.33 ± 0.02	0.11 ± 0.04	0.65 ± 0.06	66.1 ± 6.2
NASH	64 ± 9	39 ± 6	0.80 ± 0.02	0.36 ± 0.04	0.23 ± 0.03*	0.34 ± 0.05*	104.1 ± 13.4*
Silibinin	63 ± 5	31 ± 3	0.62 ± 0.05	0.26 ± 0.01**	0.15 ± 0.02	0.35 ± 0.03	86.3 ± 10.0**
ANOVA	0.709	0.204	0.097	0.102	0.0551	0.0004	0.0442

Measurements were obtained from plasma of rats in the fasting state, after 12 weeks of standard diet (control) or high-fat diet alone (NASH), or supplemented with silibinin–phosphatidylcholine complex (Silibinin), at a dose of 200 mg kg^−1^ as silibinin, during the last 5 weeks. Values are expressed as means ± SE of six rats for Control and NASH groups and of eight rats for Silibinin group. The *P*-values for the ANOVA Fisher's PLSD test are given.

For pairwise comparisons, *P < .05 versus control group and **P < .05 versus NASH group.

**Table 3:** Effect of silibinin on liver histology scoring for macrovesicular steatosis and inflammatory cells infiltration.

Groups	*N*	Steatosis^a^				Inflammation^b^			
		0	1	2	3	0	1	2	3
Control	6	5	1	0	0	5	0	1	0
NASH	6	0	0	1	5	0	0	3	3
Silibinin	8	3	2	1	2	2	1	4	1
Chi-square		*P* < .05				*P* < .05			

Histological scoring for steatosis and inflammation from rats fed a standard diet for 12 weeks (control) or a high-fat diet alone (NASH), or supplemented with silibinin-phosphatidylcholine complex (Silibinin), at a dose of 200 mg kg^−1^ as silibinin, during the last 5 weeks. The *P* values for the Chi square test are given and differences are significant among all groups for both parameters.

^a^Macrovesicular steatosis: grade 0 absent; grade 1 < 33%; grade 2: 33–66%; grade 3: >66%. ^b^Inflammation: 0 normal; 1 mild; 2 moderate; 3 severe.
